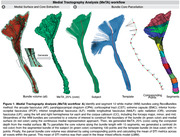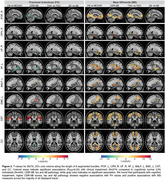# Along‐Tract Analysis of White Matter Bundles in Alzheimer’s disease using Medial Tractography Analysis (MeTA)

**DOI:** 10.1002/alz.091159

**Published:** 2025-01-09

**Authors:** Iyad Ba Gari, Siddharth Narula, Shruti Gadewar, Talia M Nir, Neda Jahanshad

**Affiliations:** ^1^ Imaging Genetics Center, Mark and Mary Stevens Neuroimaging & Informatics Institute, University of Southern California, Marina del Rey, CA USA

## Abstract

**Background:**

Along‐tract analysis of white matter (WM) bundles can help map detailed patterns of WM pathway degeneration in Alzheimer's disease. Here, we present Medial Tractography Analysis (MeTA), which aims to minimize partial voluming and microstructural heterogeneity in diffusion MRI (dMRI) metrics by extracting and parcellating the volume along the bundle length while preserving bundle shape and capturing variation within and along WM bundles. We evaluated along‐tract WM microstructure associations with clinical measures in ADNI using MeTA.

**Method:**

We used dMRI and clinical data from 714 participants (aged 55‐96 years, 52% females) enrolled in ADNI3. The dMRI data were processed using: denoised using Local PCA, deGibbs, and corrected for eddy current and motion artifacts using FSL eddy. The fiber orientation distributions (iFOD2) were estimated using a multi‐tissue constrained spherical deconvolution approach. Whole brain probabilistic streamline tractography was generated. RecoBundles was used to identify and segment 12 WM bundles. We applied MeTA to the bundles and evaluated the diffusion tensor (DTI) fractional anisotropy (FA) and mean diffusivity (MD) associations with 1) clinical impairment (N=274) compared to cognitively normal (CN); 2) Clinical Dementia Rating Scale Sum of Boxes (CDR‐SB); 3) tau load indexed with AV‐1451; and 4) amyloid (Aβ) with FBB/FBP. Full details of the pipelines can be found in Figure 1. We used linear mixed models, with age and sex as fixed effects, and scan sites as nested random variables. We corrected for multiple comparisons across 315 tests (21 bundles x 15 regions) using the false discovery rate (FDR) procedure (q=0.05).

**Result:**

Participants with cognitive impairment, higher CDR‐SB scores, tau and Aβ pathology showed negative associations with FA values and positive associations with MD measures across bundles such as IFOF_L, CPH_R, UF_R, AF_L, MdLF_L, EMC_L, CST, and CC as displayed in Figure 2. All the statistical tests were completed across 15 segments in one bundle in about 20 seconds.

**Conclusion:**

MeTA is a fast and reliable approach for identifying regional WM brain abnormalities in neurodegenerative populations and can be used to map localized effects of disease progression. MeTA is a highly scalable approach that can be applied to large‐scale collaborative multisite AD efforts and consortia.